# Photosensitizer synergistic effects: D–A–D structured organic molecule with enhanced fluorescence and singlet oxygen quantum yield for photodynamic therapy†
†Electronic supplementary information (ESI) available. See DOI: 10.1039/c7sc04694d


**DOI:** 10.1039/c7sc04694d

**Published:** 2018-01-18

**Authors:** Jianhua Zou, Zhihui Yin, Peng Wang, Dapeng Chen, Jinjun Shao, Qi Zhang, Liguo Sun, Wei Huang, Xiaochen Dong

**Affiliations:** a Key Laboratory of Flexible Electronics (KLOFE) , Institute of Advanced Materials (IAM) , Nanjing Tech University (NanjingTech) , 30 South Puzhu Road , Nanjing , 211800 , China . Email: iamxcdong@njtech.edu.cn; b Shaanxi Institute of Flexible Electronics (SIFE) , Northwestern Polytechnical University (NPU) , 127 West Youyi Road , Xi’an 710072 , China . Email: iamwhuang@njtech.edu.cn; c Department of Radiology , Binzhou Medical University Hospital , Yantai , Shandong 264100 , China . Email: zisetasong@sina.com; d School of Pharmaceutical Sciences , Nanjing Tech University (NanjingTech) , 30 South Puzhu Road , Nanjing 211800 , China

## Abstract

The development of photosensitizers with high fluorescence intensity and singlet oxygen (^1^O_2_) quantum yields (QYs) is of great importance for cancer diagnosis and photodynamic therapy (PDT). Diketopyrrolopyrrole (DPP) and boron dipyrromethene (BODIPY) are two kinds of building block with great potential for PDT. Herein, a novel donor–acceptor–donor (D–A–D) structured organic photosensitizer DPPBDPI with a benzene ring as a π bridge linking DPP and BODIPY has been designed and synthesized. The results indicate that the combination of DPP with BODIPY can simultaneously increase the fluorescence QY (5.0%) and the ^1^O_2_ QY (up to 80%) significantly by the synergistic effect of the two photosensitizers. By nanoprecipitation, DPPBDPI can form uniform nanoparticles (NPs) with a diameter of less than 100 nm. The obtained NPs not only exhibit high photo-toxicity, but also present negligible dark toxicity towards HeLa cells, demonstrating their excellent photodynamic therapeutic efficacy. *In vivo* fluorescence imaging shows that DPPBDPI NPs can target the tumor site quickly with the enhanced permeability and retention (EPR) effect and can effectively inhibit tumor growth using photodynamic therapy even with low doses (0.5 mg kg^–1^). The enhanced imaging and photodynamic performance of DPPBDPI suggest that the synergistic effect of DPP and BODIPY provides a novel theranostic platform for cancer diagnosis and photodynamic therapy.

## Abstract 2

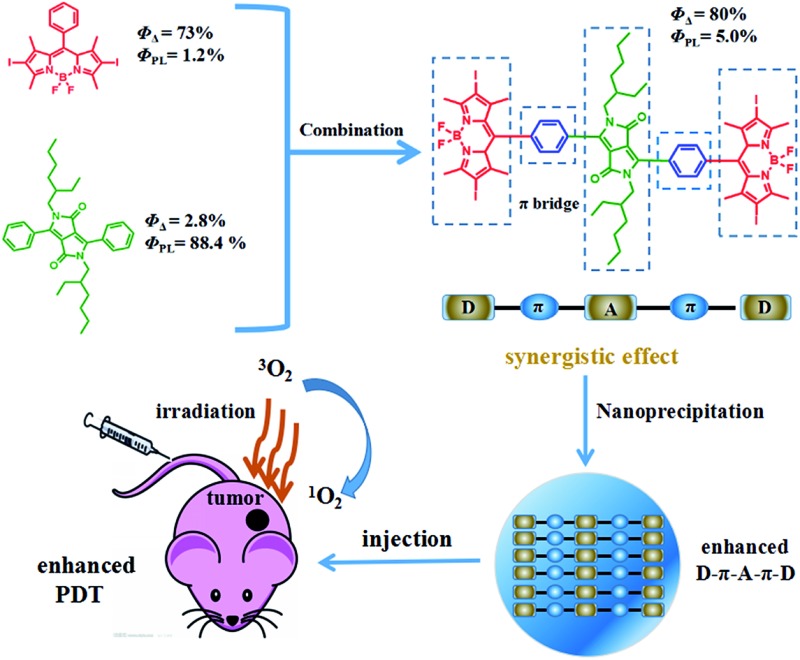
Novel photosensitizers have been developed with high ^1^O_2_ quantum yields and strong fluorescence for cancer diagnosis and PDT.

## Introduction

Cancer has become the second leading cause of death following heart disease, and has posed a great threat to the health of human beings.^1^ Traditional cancer therapeutic approaches, including surgery, chemotherapy and radiotherapy, sometimes suffer from invasion, high systemic damage, and no targeting and may inevitably destroy the immune system and result in an increased incidence of side effects.^2^–^5^ Therefore, it is essential to develop more effective approaches for cancer treatment as the global cancer morbidity rises. Photodynamic therapy (PDT), as a non-invasive and potentially effective alternative to conventional approaches, has attracted much attention over the past few decades.^6^–^15^ The key aspect of PDT is the use of an efficient photosensitizer which can convert triplet oxygen (^3^O_2_) to reactive singlet oxygen (^1^O_2_) under light irradiation. Diketopyrrolopyrrole (DPP) and boron dipyrromethene (BODIPY) derivatives are two kinds of organic dyes with strong fluorescence and photostability, which makes them potential candidates for bio-imaging.^16^–^30^ For example, Siegwart *et al.* reported a size controlled pH activable BODIPY compound, which can detect cancer precisely through a fluorescence imaging method.^31^ To enhance the fluorescence for bio-imaging, Chen *et al.* synthesized photoconversion-tunable fluorophore BODIPY vesicles for wavelength-dependent photoinduced cancer therapy.^32^ However, both suffer from low singlet oxygen quantum yields (^1^O_2_ QYs), which greatly limits their application for cancer diagnosis and photodynamic therapy.

It is well known that heavy atoms such as bromine and iodine can facilitate the intersystem crossing (ISC) rate to increase the ^1^O_2_ QYs,^7^ and the BODIPY core with heavy atom incorporation can increase the singlet oxygen QY dramatically.^33^–^35^ However, previous studies indicated that two bromine substituted diketopyrrolopyrrole (DPP) derivatives could not improve the singlet oxygen QYs effectively.^36^,^37^ This phenomenon may be due to heavy atoms not enhancing the spin orbit coupling (SOC) of DPP compounds effectively. On the other hand, heavy atoms may inevitably quench the fluorescence of the photosensitizer, which is unfavourable for bio-imaging guided cancer therapy. Therefore, the design and synthesis of novel photosensitizers with high ^1^O_2_ QYs and strong fluorescence are urgent and essential both for cancer diagnosis and PDT.

**Scheme 1:**
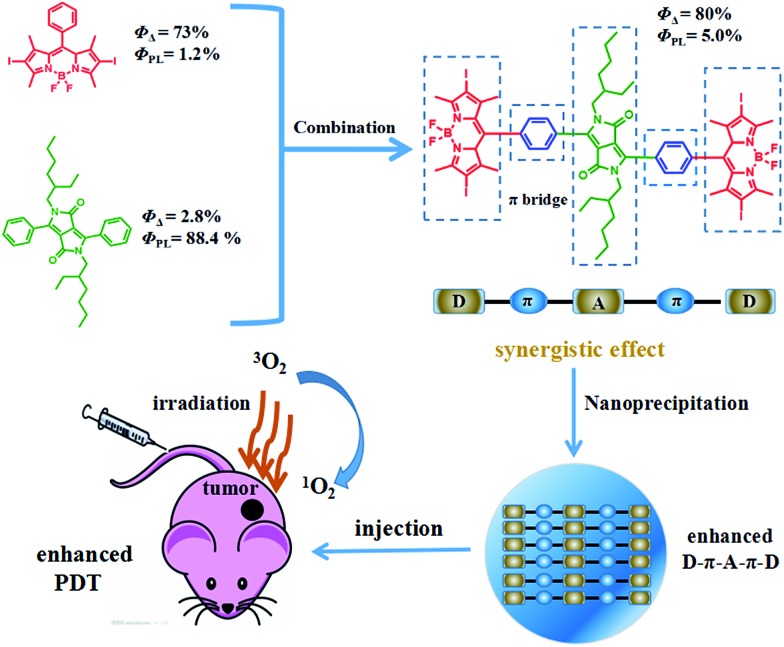
Illustration of the D–A–D structured DPPBDPI NPs with enhanced ^1^O_2_ QYs and fluorescence as a theranostic agent for PDT.

It is expected that donor–acceptor–donor (D–A–D) structured DPPBDPI, with a benzene ring as a π bridge, will combine both the advantages of DPP and BDPI. Herein, DPP was chosen to be the electron-deficient core, since this moiety allows for the control of small molecule solution processability and solid-state molecular ordering through modulation of the *N*-alkyl substituents. The DPP core has also demonstrated promising optical properties and charge carrier mobility. The introduction of π-stacking moieties (BDPI) onto the ends of DPP would facilitate end-to-end π–π interactions, leading to enhanced charge transport between adjacent molecules. As a result, fluorescence imaging guided PDT will be enhanced. ^1^O_2_ QY (*Φ*_Δ_) and fluorescence QY (*Φ*_PL_) measurements indicated that DPPBDPI exhibited a higher ^1^O_2_ QY than DPP and BDPI, respectively. This indicates that the photosensitizer synergistic effects not only overcome the fluorescence quenching caused by the heavy atoms on the BODIPY core, but they also simultaneously improve their singlet oxygen quantum yields. *In vitro* and *in vivo* experiments demonstrated that the DPPBDPI nanoparticles (NPs) obtained by nanoprecipitation possess low dark toxicity and ultra-high phototoxicity (half-maximal inhibitory concentration, IC_50_ = 0.06 μM), which can inhibit the migration of HeLa cells effectively. Furthermore, *in vivo* fluorescence imaging indicates that targeted DPPBDPI NPs can accumulate at the tumor site by the enhanced permeability and retention (EPR) effect to inhibit tumor growth without side effects at low doses (Scheme 1).

## Experimental

### Materials and equipment

All chemicals were purchased from Sigma and used without further purification. The ^1^H NMR and ^13^C NMR spectra were recorded on a Bruker DRX NMR spectrometer (500 MHz) in CDCl_3_ solution at 298 K with a solvent residual as the internal standard (CDCl_3_, *δ* = 7.26 ppm). UV-vis spectra were measured on a spectrophotometer (UV-3600 UV-Vis-NIR, Shimadzu, Japan). The fluorescence spectra were recorded on an F4600 spectrometer (HITACHI, Japan). DLS was performed with a 90 Plus particle size analyzer (Brookhaven Instruments, USA). TEM of the nanoparticles was carried out using JEOL JEM-2100 equipment. The bio-images of the tumor, heart, liver, spleen, and kidney were recorded on a PerkinElmer IVIS Lumina K.

### Preparation of DPP, BDPI and DPPBDPI nanoparticles

The nanoparticles of the three compounds were prepared by nanoprecipitation. Taking DPPBDPI as an example, 200 μL of DPPBDPI (5 mg mL^–1^) in tetrahydrofuran (THF) was added to 5 mL of water under vigorous stirring at room temperature. After the mixture was stirred for 20 min, THF was removed using nitrogen bubbling. The DPPBDPI NPs in the solution were obtained by centrifugation.

### Cell culture and MTT assay

HeLa cell lines (Institute of Biochemistry and Cell Biology, SIBS, CAS (China)) were cultured in a growth medium consisting of Dulbecco’s modified Eagle’s medium (DMEM, Gibco), supplemented with 10% fetal bovine serum under an atmosphere of 5% CO_2_ at 37 °C. Cell viability assays of the nanoparticles of the three compounds were carried out; they were first dissolved in distilled water, and were then diluted with DMEM to various concentrations and put in the 96-well plate. Then the 96-well plate was irradiated with a xenon lamp (40 mW cm^–2^) for 8 minutes. Cell viability was determined using an MTT (3-(4,5-dimethylthiazol-2-yl)-2,5-diphenyltetrazolium bromide) assay. A solution of MTT in distilled water (5 mg mL^–1^, 20 μL) was added to each well after incubation for 4 h under the same conditions at 37 °C. Then the liquid was discarded and 200 μL DMSO was added. The absorbance at 492 nm of the plate was measured on a Bio-Tek microplate reader at ambient temperature. The cell viability was then determined by the following equation: viability (%) = mean absorbance in each group incubated with different concentrations of NPs/mean absorbance in the control group.

### Cellular uptake and fluorescence imaging of cellular ROS

HeLa cells were incubated with the DPPBDPI NPs (1 μg mL^–1^, 2 mL) in a confocal dish for 24 h in the dark. Then the solution was discarded and the cells were washed with PBS (3 mL), followed by the addition of 1 mL polyoxymethylene for 25 min. Then polyoxymethylene was discarded and the cells were washed with PBS three times. The sample that was incubated with the DPPBDPI NPs for 24 h was further incubated with 10 μM of 2,7-dichlorodihydrofluorescein diacetate (DCF-DA) for another 3 min, and was washed with 1 mL PBS three times. This sample was irradiated with a xenon lamp (40 mW cm^–2^) for 3 minutes. The fluorescence images were collected using an Olympus IX 70 inverted microscope. The samples incubated with the DPPBDPI NPs for 24 h were excited at a wavelength of 540 nm and the fluorescence was collected from 550 to 600 nm. The sample incubated with DCF-DA under irradiation was excited with a 488 nm laser and the fluorescence was collected from 490 to 600 nm.

### Trypan blue staining and *in vitro* assay of 2D cell migration across an artificial gap

HeLa cells were incubated with the DPPBDPI NPs for 24 h and irradiated with a Xe lamp for 5 min (40 mW cm^–2^). After 1 h, the mother liquid was discarded and the cells were washed with PBS three times. Then a solution of trypan blue (0.6 μg mL^–1^, 100 μL) was added and after 8 minutes the images were recorded with the microscope. To investigate the ability of cell motility, an *in vitro* cell migration assay was performed. Briefly, the HeLa cells were cultured with the DPPBDPI NPs at different concentrations (0, 1, 2, 3, 4 μg mL^–1^) for 24 h, then a confluent layer of cells was wounded using a yellow tip. The open gap was then observed microscopically when the cells moved in and filled the damaged area. Micrographs were taken after wounding using an inverted microscope (Leica, German). Wound closure was measured by showing the distances between the sides of the wound.

### *In vivo* tumor treatment histology examination and bio-imaging

Animal ethical approval was obtained from the Animal Centre of Nanjing Medical University (NJMU, Nanjing, China) for a pharmacokinetic study (SCXK-2012-004). 15 nude mice were purchased and then were injected in the armpit with HeLa cells as the tumor source. When the tumor volume reached about 100 mm^3^, the mice were randomly divided into 3 groups. Groups I and II were injected in the tail vein with DPPBDPI NPs (100 μg mL^–1^, 100 μL) in PBS solution. Similarly, group III was injected with saline in the same way as the control group. After 24 h, the tumors of the control and illumination groups were irradiated with a xenon lamp for 8 minutes while the mice in the no illumination group were not irradiated. The process above was conducted for twenty days, and the tumor volume and body weight of the mice were recorded every two days. The nude mice were killed, which was followed by histological analysis. The main organs (heart, liver, spleen, lung, and kidney) and the tumor from each mouse were isolated and fixed in 4% formaldehyde solution. After dehydration, the tissues were embedded in paraffin cassettes and stained with hematoxylin and eosin (H&E), and the images were recorded on a microscope.

## Results and discussion

### Preparation and characterization of DPPBDPI

**Fig. 1:**
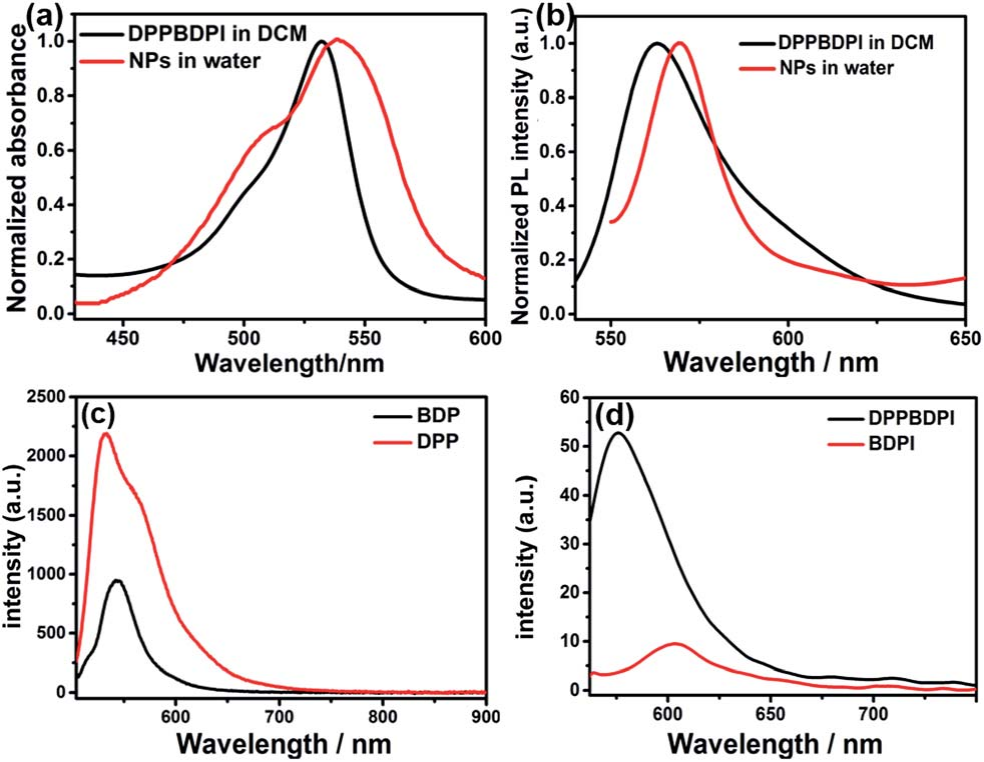
(a and b) Normalized absorption and emission spectra of DPPBDPI in DCM and NPs in water. (c) Emission spectra of DPP and BDP in DCM, showing absolute PL quantum yields of 88.4% and 24.7%, respectively. (d) Emission spectra of BDPI and DPPBDPI, showing absolute PL quantum yields of 1.2% and 5.0%, respectively.

The preparation of BDPI was described in our previous work.^38^ The synthetic procedures of DPP and DPPBDPI are proposed in the ESI.† All compounds were prepared in moderate yields (Fig. S1†). DPP, BDPI and DPPBDPI in DCM show absorption with maximum intensities of 464, 533 and 535 nm, respectively (Fig. 1a and S2†). In the case of the DPP, BDPI and DPPBDPI nanoparticles, red shifts of 6, 5 and 5 nm were observed, respectively, which were caused by aggregation in the nanoparticles. DPP, BDPI and DPPBDPI show emissions with maximum intensity at 528, 564 and 568 nm, respectively, while red shifts of 6, 18 and 10 nm were found for their nanoparticles, respectively.

High fluorescence is crucial for a photosensitizer (PS) to be used as an agent for cell imaging and diagnosis. The absolute photoluminescence (PL) quantum yields (*Φ*_PL_) of DPP, BDPI and DPPBDPI were measured. BDP has a high *Φ*_PL_ of 24.7%. When iodine atoms are introduced into the pyrrole rings, BDPI shows a sharp decrease in the PL quantum yield, to only 1.2%, which can be explained by the heavy atom effect. It is supposed that DPP, with a high *Φ*_PL_ of 88.4%, can compensate for the decreased *Φ*_PL_ when it is conjugated with BDPI to form a large conjugated D–A–D system. It can be concluded that the D–A–D structured DPPBDPI, with a benzene ring as a π bridge for electron transfer, shows a 4 times higher PL quantum yield (5.0%) compared to BDPI, which may be attributed to the synergistic effect of the two PSs.

**Fig. 2:**
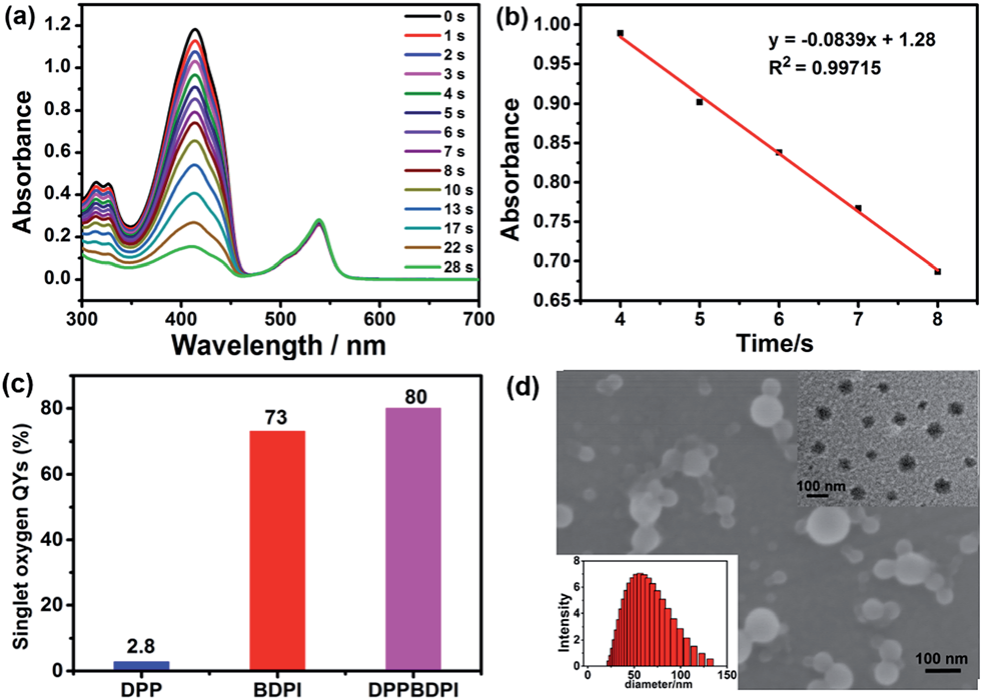
(a) The degradation of DPBF under the presence of DPPBDPI in DCM and xenon lamp irradiation. (b) Linear fitting of the absorption and the irradiation time. (c) Singlet oxygen QYs of DPP, BDPI and DPPBDPI. (d) SEM, TEM and DLS of DPPBDPI NPs, showing the size distribution from approximately 30 to 130 nm.

Furthermore, high singlet oxygen quantum yields can promise high photo-toxicity, which is fundamental to PDT. The singlet oxygen QYs of DPP, BDPI and DPPBDPI were measured using 1,3-diphenylisobenzofuran (DPBF) as a probe and MB (methylene blue) as the standard. The absorbance of DPBF at 414 nm was recorded for different irradiation times. The singlet oxygen QY was calculated according to the literature.^35^ As shown in Fig. 2a, DPBF degrades at a considerably high speed under the presence of DPPBDPI, while BDPI degrades at a lower speed. DPP shows the lowest speed of degradation (Fig. S3†). DPP shows an almost negligible singlet oxygen QY (2.8%), while BDPI shows a much higher QY (73%). This phenomenon can be explained by the so called heavy atom effect. For DPPBDPI, a higher singlet oxygen QY was observed (up to 80%). It can be found that the singlet oxygen QY of DPPBDPI is about 29-fold that of DPP and a little higher than that of BDPI, indicating that two BDPI cores can compensate for the low singlet oxygen QY of DPP. It can be observed that combination of DPP with BDPI can enhance the singlet oxygen quantum yield of DPPBDPI.

Since the three compounds can hardly be dissolved in water, to resolve the solubility of the three compounds, nanoprecipitation was used to improve their water solubility. In general, 10 mg of DPP, BDPI and DPPBDPI was dissolved in THF, respectively, and 200 μL of this solution was injected into 10 mL distilled water with stirring. After being purged with nitrogen for 20 min to drive the THF off, the concentration of the obtained solution is 200 μg mL^–1^. Scanning electron microscopy (SEM), transmission electron microscopy (TEM) and dynamic light scattering (DLS) were used to characterize the morphology and diameter of the DPPBDPI nanoparticles. As shown in Fig. 2c and d, DPPBDPI is able to self-assemble and form spherical nanoparticles with size distribution from approximately 30 to 130 nm.

### MTT assay, cellular uptake, ROS generation and cell migration *in vitro*

**Fig. 3:**
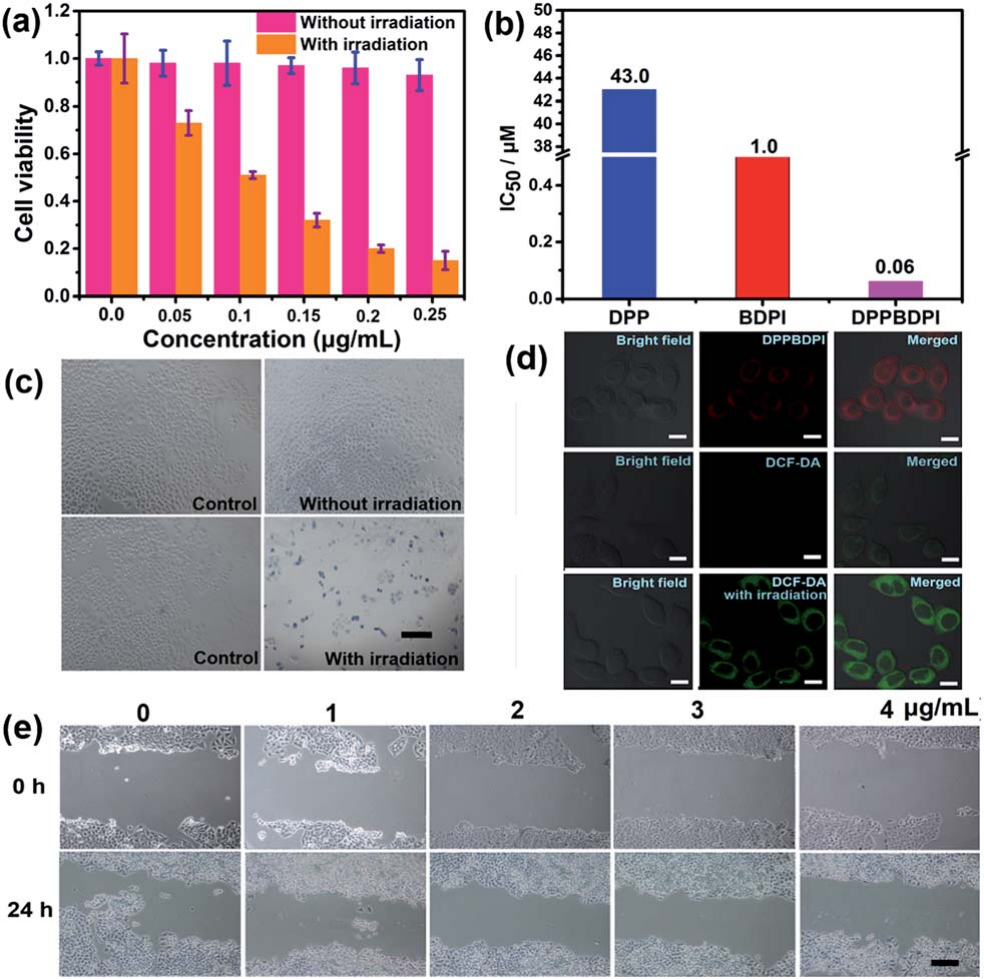
(a) MTT assay of DPPBDPI NPs on HeLa cells, showing IC_50_ of 0.1 μg mL^–1^ (0.06 μM). (b) IC_50_ of DPP, BDPI and DPPBDPI NPs on HeLa cells. (c) Trypan blue stained HeLa cells with DMEM or DPPBDPI NP incubation exposed to a Xe lamp, blue indicates dead cells. Scale bar: 25 μm. (d) Cellular uptake of DPPBDPI NPs in HeLa cells, ROS generation in HeLa cells with DCF-DA as a probe without or with excitation at 488 nm. Scale bar: 10 μm. (e) Migration of HeLa cells incubated with different concentrations of DPPBDPI NPs (0, 1, 2, 3 and 4 μg mL^–1^). Scale bar: 25 μm.

High phototoxicity upon light irradiation as well as low dark toxicity is highly essential for phototherapy to minimize side effects and enhance the therapeutic efficiency. The MTT assay in Fig. 3a shows that the DPPBDPI NPs have the lowest phototoxicity half-maximal inhibitory concentration (IC_50_, 0.1 μg mL^–1^, 0.06 μM). The BDPI NPs (with moderate phototoxicity, 0.6 μg mL^–1^, 1 μM) and the DPP NPs (with highest phototoxicity, 22 μg mL^–1^, 43.0 μM) are perfectly consistent with the singlet oxygen QYs (Fig. 3b and S4†). In addition, cell viability of the group incubated with the three nanoparticles without irradiation remained high, indicating the low dark toxicity of these nanoparticles. To summarize, although incorporation of heavy atoms into the BODIPY core decreases the fluorescence of the BDPI, conjugation of DPP with BDPI can enhance the fluorescence of BDPI greatly. In return, BDPI is able to compensate for the low singlet oxygen QY of DPP. With the benzene ring as a π bridge, the enhanced D–A–D structured DPPBDPI with a large conjugated system exhibits both enhanced singlet oxygen QY and fluorescence, which makes it suitable for both diagnosis and PDT.

The PDT efficiency of DPPBDPI NPs *in vitro* was further investigated using trypan blue staining. Cells in the control groups are colorless, confirming that irradiation alone or DPPBDPI NPs incubated alone are harmless to the cells. However, most cells incubated with DPPBDPI NPs were killed upon laser irradiation, as indicated by the intense homogeneous blue color (Fig. 3c).^39^

To investigate the application of DPPBDPI as an agent for cell imaging, the cellular uptake of DPPBDPI NPs is shown in Fig. 3d. Red fluorescence can be observed, indicating that DPPBDPI NPs can be used for cell imaging *in vitro*. 2′,7′-dichlorofluorescein diacetate (DCF-DA) was used as the probe for singlet oxygen detection in cells. Very weak green fluorescence can be detected when HeLa cells are not irradiated, implying DPPBDPI NPs alone cannot generate ROS without light irradiation. However, bright green fluorescence can be observed upon excitation at 488 nm, which indicates that DPPBDPI NPs are able to generate strong singlet oxygen under irradiation.

It is essential for a photosensitizer to inhibit cell migration because HeLa tumors may migrate *in vivo*. Therefore, the cell migration of the NPs has been investigated. As shown in Fig. 3e, in the control group, HeLa cells efficiently moved in and filled the open gap at 24 h after wounding. However, the increased wound closure was greatly suppressed in the presence of the DPPBDPI NPs even at a low concentration (1 μg mL^–1^, 0.6 μM). These results show that DPPBDPI NPs can effectively inhibit the migration of HeLa cells, showing their potential to inhibit the transfer of tumors *in vivo*.

### Fluorescence imaging and photodynamic therapy *in vivo*

**Fig. 4:**
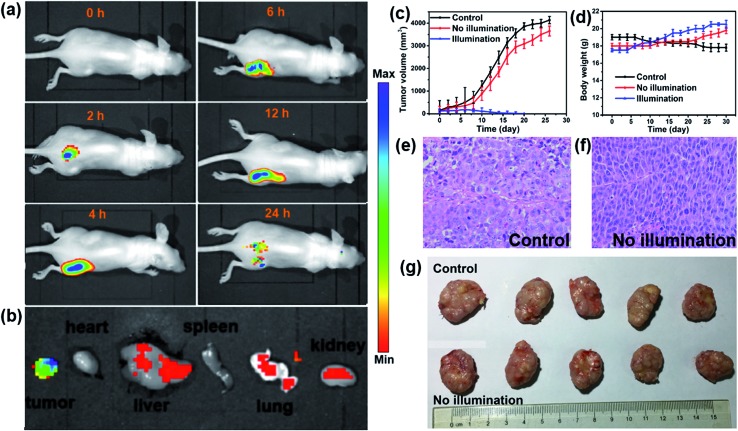
(a) *In vivo* time dependent fluorescence imaging. (b) Bio-distribution of DPPBDPI NPs in the tumor, heart, liver, spleen, lung and kidney after injection for 24 h. (c) Tumor volume change during treatment over a month. (d) The body weight change reported every two days. (e and f) H&E-stained images of the tumor histologic section for the control and no illumination groups. (g) Pictures of the tumors of the sacrificed mice after treatment.

*In vivo* fluorescence images of tumor tissues before and after DPPBDPI NP (100 μg mL^–1^, 100 μL) tail injection (*i.v.*) of under 540 nm laser irradiation were recorded at different times. The clear and strong fluorescence signals of the tumors shown in these images suggest that DPPBDPI NPs can efficiently accumulate at tumor sites owing to the enhanced permeability and retention (EPR) effect. As shown in Fig. 4a, at 4 h post injection, the fluorescence signal intensity reached a maximum degree, which illustrates that 4 h after injection was the optimal time for PDT. In addition, the fluorescence signal at tumor sites after 24 h post injection was still higher than that of pre-injection of the NPs, indicating that DPPBDPI NPs can serve as long-term fluorescence imaging agents. Afterwards, the mice were sacrificed and the bio-distribution indicates that DPPBDPI NPs mainly stay in the tumor, lung and kidney (Fig. 4b).^43^

**Fig. 5:**
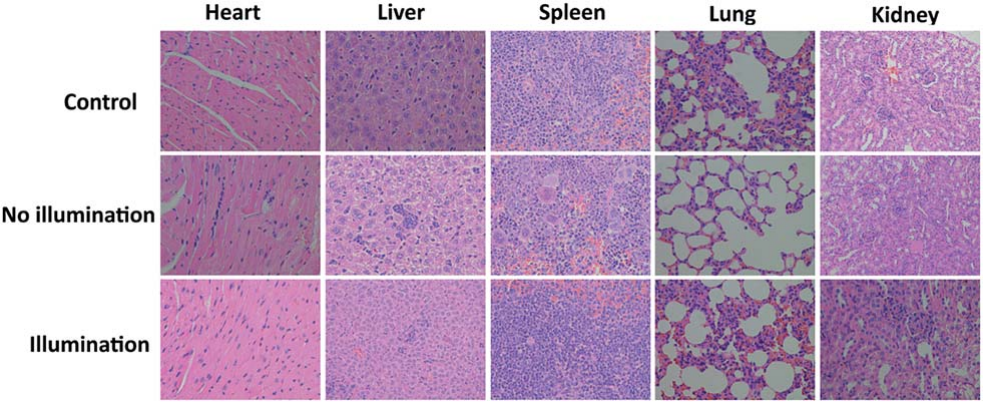
H&E stained images of the heart, liver, spleen, lung, and kidney of control, no illumination and illumination groups, respectively.

To further investigate the PDT efficacy of DPPBDPI NPs *in vivo*, 15 nude mice bearing HeLa tumors are divided into three groups at random. When the tumor volume reaches about 100 mm^3^, 10 μg (100 μg mL^–1^, 100 μL) of DPPBDPI NPs was injected into the mice *via* the tail vein in the no illumination and illumination groups, while the control group was injected with PBS. In the case of the illumination group, the five mice were irradiated after injection for 4 h. The tumor volume and body weight were recorded every two days. As shown in Fig. 4c, the tumor volume of the control group and the no illumination group increases at a high speed, while that of the illumination group remains unchanged, which indicates that DPPBDPI has high phototoxicity for the tumor. After administering treatment 10 times (the 20^th^ day), the tumor of the illumination group disappears and although the mice were still kept for another 6 days, no obvious tumors were observed, suggesting that the DPPBDPI NPs have high efficiency for tumor treatment. In regards to the control group, the weight of the mice gradually decreases while for the no illumination and illumination groups, the mice become fatter, indicating the low dark toxicity of DPPBDPI NPs (Fig. 4d). Images of the mice after treatment are shown in Fig. S5.† Mice were killed after treatment and the tumors are shown in Fig. 4g. Hematoxylin and eosin (H&E)-stained images of the tumor histologic section of the control and no illumination groups are shown in Fig. 4e and f, and the nuclei of the cells remain almost unchanged while the tumors of the illumination group disappear, suggesting the low dark toxicity of DPPBDPI NPs. All in all, DPPBDPI NPs can inhibit tumor growth effectively without causing damage to main organs (heart, liver, spleen, lung, and kidney) (Fig. 5), suggesting their good bio-compatibility.^40^–^42^

## Conclusions

In summary, a D–A–D structured photosensitizer DPPBDPI was designed and synthesized. The results show that BDPI is able to compensate for the low singlet oxygen QY of DPP, while DPP can improve the fluorescence of BDPI further. DPPBDPI, with a high singlet oxygen QY (80%) and an ultra-low phototoxicity IC_50_ of 0.06 μM on the HeLa cells, shows low dark toxicity, high phototoxicity and excellent bio-compatibility. It is confirmed that combination of DPP with BDPI makes DPPBDPI exhibit enhanced fluorescence and singlet oxygen QYs for cell imaging guided PDT according to ‘photosensitizer synergistic effects’.

## Conflicts of interest

There are no conflicts to declare.

## Supplementary Material

Supplementary information

## References

[cit1] Siegel R. L., Miller K. D., Jemal A. (2016). Ca-Cancer J. Clin..

[cit2] Shivran N., Tyagi M., Mula S., Gupta P., Saha B., Patro B. S., Chattopadhyay S. (2016). Eur. J. Med. Chem..

[cit3] Laine M., Barbosa N. A., Kochel A., Osiecka B., Szewczyk G., Sarna T., Ziółkowski P., Wieczorek R., Filarowski A. (2017). Sens. Actuators, B.

[cit4] Song X., Chen Q., Liu Z. (2014). Nano Res..

[cit5] Carija A., Puizina-Ivic N., Vukovic D., Miric Kovacevic L., Capkun V. (2016). Photodiagn. Photodyn. Ther..

[cit6] Boens N., Leen V., Dehaen W. (2012). Chem. Soc. Rev..

[cit7] Kamkaew A., Lim S. H., Lee H. B., Kiew L. V., Chung L. Y., Burgess K. (2013). Chem. Soc. Rev..

[cit8] Li M., Gao Y., Yuan Y. Y., Wu Y. Z., Song Z. F., Tang B. Z., Liu B., Zheng Q. C. (2017). ACS Nano.

[cit9] Xu S. D., Yuan Y. Y., Cai X. L., Zhang C. J., Hu F., Liang J., Zhang G. X., Zhang D. Q., Liu B. (2015). Chem. Sci..

[cit10] Cai X. L., Zhang C. J., Wei Lim F. T., Chan S. J., Bandla A., Chuan C. K., Hu F., Xu S. D., Thakor N. V., Liao L. D., Liu B. (2016). Small.

[cit11] Gu B. B., Wu W. B., Xu G. X., Feng G. X., Yin F., Joo Chong P. H., Qu J. L., Yong K. T., Liu B. (2017). Adv. Mater..

[cit12] Wang S. H., Shang L., Li L. L., Yu Y. J., Chi C. W., Wang K., Zhang J., Shi R., Shen H. Y., Waterhouse G. I. N., Liu S. J., Tian J., Zhang T. R., Liu H. Y. (2016). Adv. Mater..

[cit13] Zhou J. F., Gai L. Z., Zhou Z. K., Yang W., Mack J., Xu K. J., Zhao J. Z., Zhao Y., Qiu H. L., Chan K. S., Shen Z. (2016). Chem.–Eur. J..

[cit14] Lincoln R., Kohler L., Monro S., Yin H. M., Stephenson M., Zong R. F., Chouai A., Dorsey C., Hennigar R., Thummel R. P., McFarland S. A. (2013). J. Am. Chem. Soc..

[cit15] Jin G. R., Feng G. X., Qin W., Tang B. Z., Liu B., Li K. (2016). Chem. Commun..

[cit16] Kim K. H., Jung D. H., Kim D., Lee A., Choi K., Kim Y., Choi S. H. (2011). Dyes Pigm..

[cit17] Luňák S., Vala M., Vyňuchal J., Ouzzane I., Horáková P., Možíšková P., Eliáš Z., Weiter M. (2011). Dyes Pigm..

[cit18] Luňák S., Vyňuchal J., Vala M., Havel L., Hrdina R. T. (2009). Dyes Pigm..

[cit19] Lan M., Zhang J., Zhu X., Wang P., Chen X., Lee C. S., Zhang W. (2015). Nano Res..

[cit20] Xu J., Sun S., Li Q., Yue Y., Li Y., Shao S. (2015). Analyst.

[cit21] Turan I. S., Yildiz D., Turksoy A., Gunaydin G., Akkaya E. U. (2016). Angew. Chem., Int. Ed. Engl..

[cit22] Meng L. B., Zhang W., Li D., Li Y., Hu X. Y., Wang L., Li G. (2015). Chem. Commun..

[cit23] Guo S., Xu L., Xu K., Zhao J., Küçüköz B., Karatay A., Yaglioglu H. G., Hayvali M., Elmali M. (2015). Chem. Sci..

[cit24] Tian J., Zhou J., Shen Z., Ding L., Yu J. S., Ju H. A. (2015). Chem. Sci..

[cit25] Umezawa K., Matsui A., Nakamura Y., Citterio D., Suzuki K. B. (2009). Chemistry.

[cit26] Wang T., Hou Y., Chen Y., Li K., Cheng X., Zhou Q., Wang X. (2015). Dalton Trans..

[cit27] Palao E., Slanina T., Muchova L., Solomek T., Vitek L., Klan P. (2016). J. Am. Chem. Soc..

[cit28] Wang J., Lu Y., McGoldrick N., Zhang C., Yang W., Zhao J., Draper S. M. (2016). J. Mater. Chem. C.

[cit29] He J., Wang Y., Missinato M. A., Onuoha E., Perkins L. A., Watkins S. C., St Croix C. M., Tsang M., Bruchez M. P. (2016). Nat. Methods.

[cit30] Cakmak Y., Kolemen S., Duman S., Dede Y., Dolen Y., Kilic B., Kostereli Z., Yildirim L. T., Dogan A. L., Guc D., Akkaya E. U. (2011). Angew. Chem., Int. Ed. Engl..

[cit31] Xiong H., Zuo H., Yan Y. F., Occhialini G., Zhou K. J., Wan Y. H., Siegwart D. J. (2017). Adv. Mater..

[cit32] He H., Ji S. S., He Y., Zhu A. J., Zou Y. L., Deng Y. B., Ke H. T., Yang H., Zhao Y. L., Guo Z. Q., Chen H. B. (2017). Adv. Mater..

[cit33] Liu X., Wu M., Hu Q., Bai H., Zhang S., Shen Y., Tang G., Ping Y. (2016). ACS Nano.

[cit34] Huang L., Li Z., Zhao Y., Zhang Y., Wu S., Zhao J., Han G. (2016). J. Am. Chem. Soc..

[cit35] Shi H. X., Sun W. C., Liu C. B., Gu G. Y., Ma B., Si W. L., Fu N. N., Zhang Q., Huang W., Dong X. C. (2016). J. Mater. Chem. B.

[cit36] Cai Y., Tang Q. Y., Wu X. J., Si W. L., Zhang Q., Huang W., Dong X. C. (2016). ACS Appl. Mater. Interfaces.

[cit37] Cai Y., Tang Q. Y., Wu X. J., Si W. L., Huang W., Zhang Q., Dong X. C. (2016). ChemistrySelect.

[cit38] Zou J. H., Yin Z. H., Ding K. K., Tang Q. Y., Li J. W., Si W. L., Shao J. J., Zhang Q., Huang W., Dong X. C. (2017). ACS Appl. Mater. Interfaces.

[cit39] Tian Q. W., Tang M. H., Sun Y. G., Zou R. J., Chen Z. G., Zhu M. F., Yang S. P., Wang J. L., Wang J. H., Hu J. Q. (2011). Adv. Mater..

[cit40] Cheng L., Kamkaew A., Sun H. Y., Jiang D. W., Valdovinos H. F., Gong H., England C. G., Goel S., Barnhart T. E., Cai W. B. (2016). ACS Nano.

[cit41] Li M., Teh C., Ang C. Y., Tan S. Y., Luo Z., Qu Q., Zhang Y., Korzh V., Zhao Y. (2015). Adv. Funct. Mater..

[cit42] Li W., Rong P., Yang K., Huang P., Sun K., Chen X. (2015). Biomaterials.

[cit43] Miao Q., Lyu Y., Ding D., Pu K. (2016). Adv. Mater..

