# Supplementary material for: Photosensitizer synergistic effects: D–A–D structured organic molecule with enhanced fluorescence and singlet oxygen quantum yield for photodynamic therapy
Source: Chem Sci. 2018 Jan 18;9(8):2188–94. doi: 10.1039/c7sc04694d (PMC5903368; doi:10.1039/c7sc04694d)
Supplement: Supplementary file 1 [file SC-009-C7SC04694D-s001.pdf]

Electronic Supplementary Material (ESI) for Chemical Science.
This journal is © The Royal Society of Chemistry 2018

Supporting Information

Photosensitizers Synergistic Effect: D-A-D Structured Organic Molecule with Enhanced Fluorescence and Singlet Oxygen Quantum Yield for Photodynamic Therapy

Jianhua Zou,[a] Zhihui Yin[a], Peng Wang[a], Dapeng Chen[a], Jinjun Shao[a], Qi Zhang[d], Liguo Sun[c], Wei Huang[b*], Xiaochen Dong[a*]*

[a]Key Laboratory of Flexible Electronics (KLOFE) & Institute of Advanced Materials (IAM), Nanjing Tech University (NanjingTech), 30 South Puzhu Road, Nanjing, 211800, China.

E-mail: iamxcdong@njtech.edu.cn

[b]Shaanxi Institute of Flexible Electronics (SIFE), Northwestern Polytechnical University (NPU), 127 West Youyi Road, Xi'an 710072, China

E-mail: iamwhuang@njtech.edu.cn

[c]Department of Radiology, Binzhou Medical University Hospital, Yantai, Shandong, 264100, China.

E-mail: zisetasong@sina.com

[d]School of Pharmaceutical Sciences, Nanjing Tech University (NanjingTech), 30 South Puzhu Road, Nanjing 211800, China.

Supporting Information

Experimental

Synthesis of DPP

(2,5-bis(2-ethylhexyl)-3,6-diphenylpyrrolo[3,4-c]pyrrole-1,4(2H,5H) -dione)

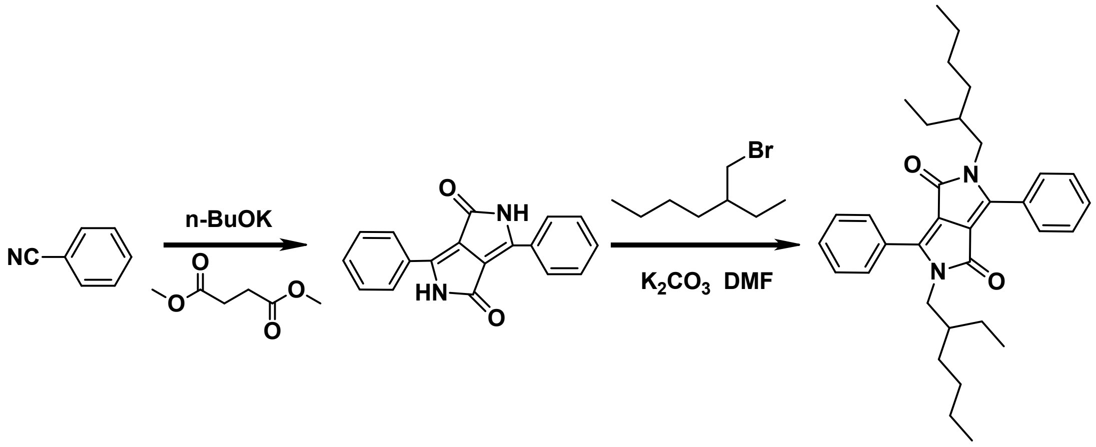

3,6-dithiophen-2-yl-2,5-dihydro-pyrrolo[3,4-c]pyrrole-1,4-dione (0.288 g, 1.0 mmol), anhydrous K_2CO_3 (0.207 g, 1.5 mmol), and 3-(bromomethyl)heptane (0.494 g, 2.4 mmol) are added into 15 mL N,N-dimethylformamide under N_2 atmosphere. The mixture is stirred at 125 °C for 24 h, then poured into 150 mL water and extracted with dichloromethane. The organic layer is washed with brine and dried with anhydrous sodium sulfate. The solvent is removed by rotary evaporation, and the crude product is purified by column chromatography (silica gel, DCM/PE = 1:2, v/v). (0.265 g, yield: 52%). ^{1}H NMR ($CDCl_3$, 400 MHz): δ (ppm) 7.67 (m, 10H), 3.70 (t, 4H), 1.00-1.50 (m, 24H), 0.70-1.0 (m, 6H). ^{13}C NMR (100 MHz, CD_2Cl_2): δ 162.15, 147.08, 132.06, 130.20, 130.19, 130.17, 130.16, 127.20, 125.39, 41.57, 31.72, 31.71, 31.70, 31.69, 29.26, 29.06, 28.94, 26.56, 22.60, 13.85, 13.84, 13.84, 13.83. MS (ESI): calcd. m/z = 512.35; found m/z = 512.45.

Synthesis of DPPBDPI

(10,10'-((2,5-bis(2-ethylhexyl)-3,6-dioxo-2,3,5,6-tetrahydropyrrolo[3,4-]yrrole-,-diyl)is4,

-phenylene))bis(5,5-difluoro-2,8-diiodo-1,3,7,9-tetramethyl-5H-dipyrrolo[1,2-

c:2’1’f][1,3,2]diazaborinin-4-ium-5-uide)

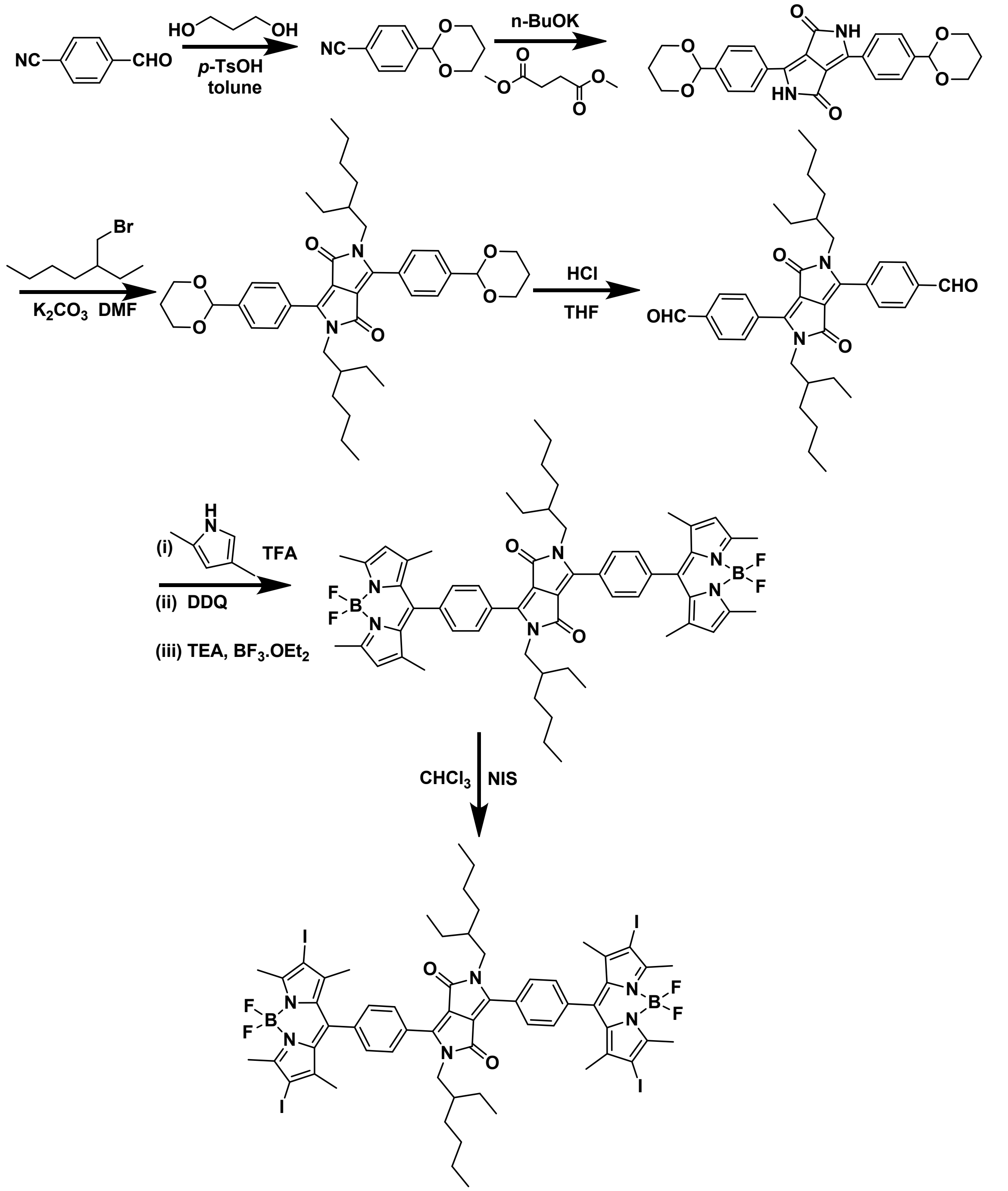

Fig. S1 Synthetic routine of DPPBDPI

Synthesis of 4-(1,3-dioxan-2-yl)benzonitrile (1)

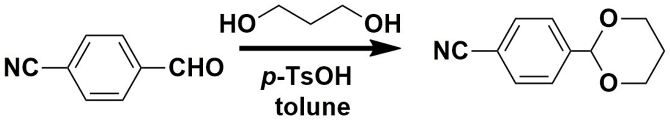

4-formylbenzonitrile (8 g, 0.06 mol), 1,3-propanediol (13 g, 0.17 mol), *p*-toluenesulfonic acid (1.04 g, 0.006 mol) were dissolved in 200 mL toluene, and the mixture were heated at 60 °C with stirring for 6 h. The solution was washed with saturated $NaHCO_3$ for three times (100 mL), the solvent was evaporated under reduced pressure and washed with water three times. Yield: 11.1 g, 96% based on 4-formylbenzonitrile. ^{1}H NMR (500 MHz, $CDCl_3$) δ 7.47-7.27 (m, 5H), 2.55 (s, 6H), 1.38 (s, 6H); ^{13}C NMR ($CDCl_3$, 500 MHz) δ 153.94, 142.08, 140.59, 134.38, 130.39, 129.51, 129.39, 127.77, 124.42, 123.93, 120.60, 119.08, 111.80, 31.40, 30.18, 29.66, 13.61, 11.01; MS (ESI): calcd. m/z = 481.97; found m/z = 481.90.

Synthesis of 3,6-bis(4-(1,3-dioxan-2-yl)phenyl)pyrrolo[3,4-c]pyrrole-1,4(2H,5H)-dione (2)

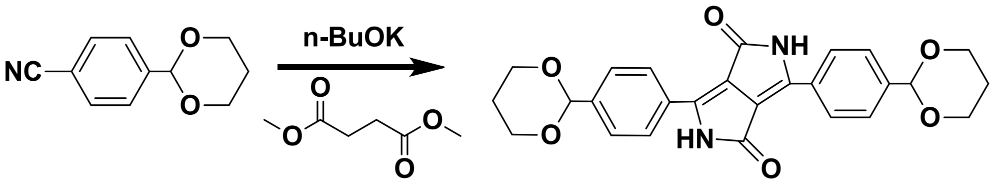

Under N_2 atmosphere, n-BuOK (11.2 g, 0.1 mol) was dissolved in 2-methyl-2-butanol (100 mL) and heated to 100 °C, then dimethyl succinate (5.84 g, 0.04 mol) was slowly dropped to solution for 15 min. After 2 h, the reaction system was cooled to 60 °C. Then a mixture of MeOH (50 mL) and AcOH (5 mL) was added to the solution to stop this reaction. The mixture was filtered and the solid was washed with distilled water (100 mL) for 4 times. The solid was dried under 50 °C. Yield: 5.8 g, 32% based on 4-(1,3-dioxan-2-yl)benzonitrile. The

product was used without further purification. The compound is poorly soluble in common organic solvents.

Synthesis of 3,6-bis(4-(1,3-dioxan-2-yl)phenyl)-2,5-di(octan-3-yl)pyrrolo[3,4-c]pyrrole-1,4(2H,5H)-dione (3)

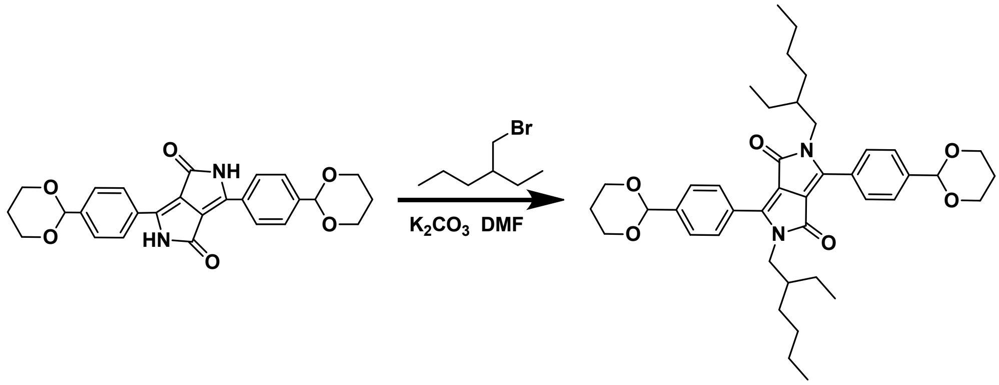

A mixture of compound 2 (4.6 g, 0.01 mol) and K_2CO_3 (3 g, 0.022 mol) was dissolved in 50 mL DMF and the solution was heated to 120 °C for 1 h. Then a mixture of 2-ethylhexyl bromide (4.25 g, 0.022 mol) and 20 mL DMF was slowly dropped into the solution. After 24 h, the solution was cooled to room temperature and poured into brine, then extracted with CH_2Cl_2 three times (100 mL). The organic layer was then washed with water and brine, dried with anhydrous sodium sulfate. The solvent was removed by evaporation, and further purified by column chromatography (silica gel, PE/DCM = 1:1, v/v). Yield: 2.8 g, 42% based on compound **2**. ^{1}H NMR (500 MHz, $CDCl_3$) δ 7.69-7.70 (d, 4H), 7.63-7.64 (d, 4H), 4.30-4.34 (d, 4H), 4.01-4,06 (t, 8H), 2.24-2.28 (q, 4H); 1.51-1.60 (m, 30H); ^{13}C NMR ($CDCl_3$, 500 MHz) δ 153.94, 142.08, 140.59, 134.38, 130.39, 129.51, 129.39, 127.77, 124.42, 123.93, 120.60, 119.08, 111.80, 31.40, 30.18, 29.66, 13.61, 11.01; MS (ESI): calcd. m/z = 684.41; found m/z = 684.90.

Synthesis of 4,4'-(2,5-di(octan-3-yl)-3,6-dioxo-2,3,5,6-tetrahydropyrrolo[3,4-c]pyrrole-1,4-diyl)dibenzaldehyde (4)

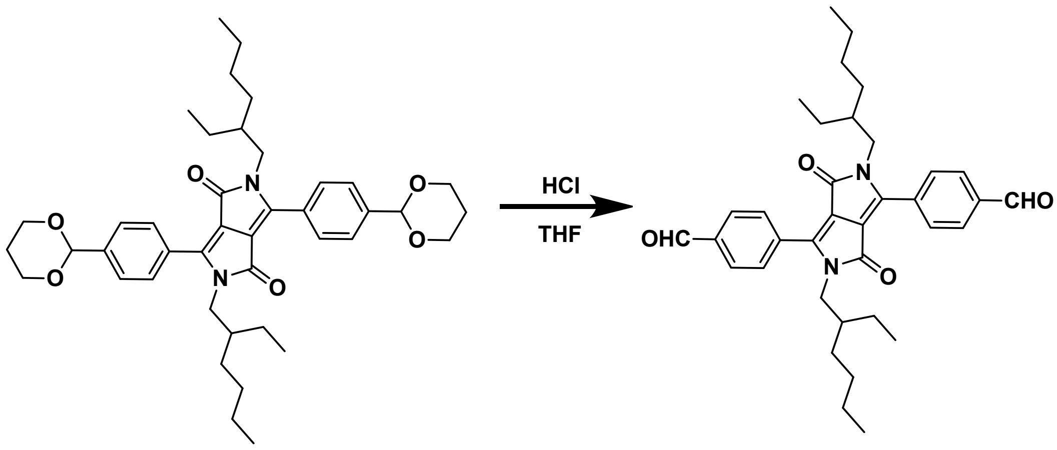

Compound **3** (1.368 g, 0.002 mol) was dissolved in a mixture of THF (40 mL) and 0.1 M HCl (10 mL), which was heated at 90 °C for 6 h. After that, the solution was cooled to room temperature and poured to brine. CH_2Cl_2 was used to extract three times (100 mL) and the organic layer was washed with saturated $NaHCO_3$ three times (100 mL). The organic layer was dried with anhydrous sodium sulfate. The solvent was removed by evaporation under reduced pressure. The obtained solid was used without further purification.

Synthesis of (10,10'-((2,5-bis(2-ethylhexyl)-3,6-dioxo-2,3,5,6- tetrahydropyrrolo[3,4-c]pyrrole-1,4-diyl)bis(4,1-phenylene))bis(5,5-difluoro-1,3,7,9-tetramethyl-5H-dipyrrolo[1,2-c:2',1'-f][1,3,2]diazaborinin-4-ium-5-uide)

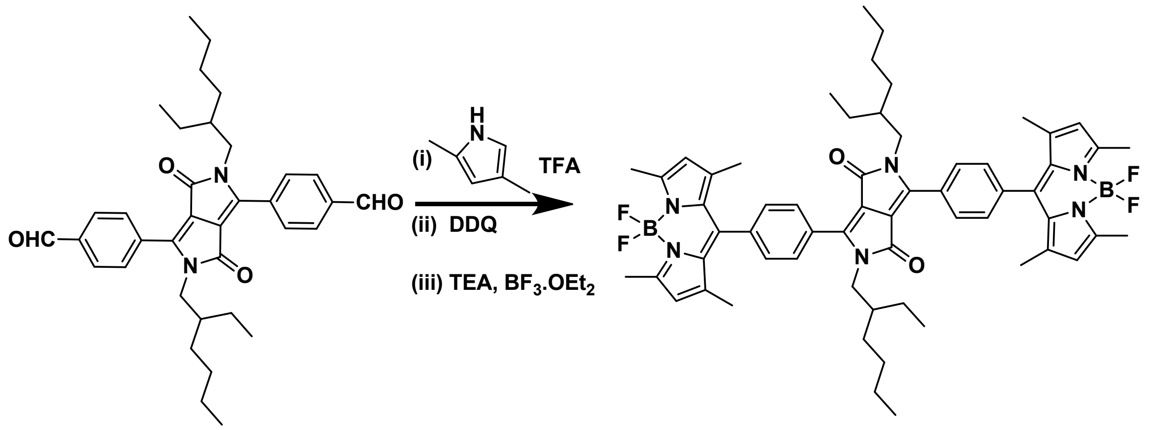

Compound 4 (0.568 g, 1 mmol) was dissolved in 50 mL DCM and put in a 100 mL three-neck flask. 2,4-dimethylporrole (0.209 g, 2.2 mmol), one drop of trifluoroacetic acid was added to the solution under the protection of N_2, which was reacted at 25 °C overnight. Then the system was put in an ice bath, followed by the addition of 10 mL NEt_3 and 10 mL $BF_3 \cdot OEt_2$. After stirring for 1 h, the reaction mixture was washed three times with water and dried over Na_2SO_4. The solvent was evaporated and the residue was purified by column chromatography on silica gel using CH_2Cl_2/PE (v/v = 1:2) as the eluent. ^{1}HNMR (500 MHz, $CDCl_3$) δ 7.63-7.70 (m, 8H), 6.00 (s, 4H), 3.50 (t, 4H), 2.58 (s, 12H); 1.60-1.51 (m, 36H), 0.98-0.70 (m, 6H); ^{13}C NMR ($CDCl_3$, 500 MHz) δ 161.52, 144.04, 142.28, 140.72, 139.41, 138.22, 136.66, 134.85, 132.06, 130.20, 130.19 130.96, 126.34, 125.94, 118.64, 109.25, 46.15, 39.40, 30.46, 29.68, 28.60, 23.62, 23.07, 14.02, 10.61.; MS (ESI): calcd. m/z= 1004.57; found m/z = 1004.90.

Synthesis of (10,10'-((2,5-bis(2-ethylhexyl)-3,6-dioxo-2,3,5,6- tetrahydropyrrolo[3,4-c]pyrrole-1,4-diyl)bis(4,1-phenylene))bis(5,5-difluoro-2,8-diiodo-1,3,7,9-tetramethyl-5H-dipyrrolo[1,2-c:2',1'-f][1,3,2]diazaborinin-4-ium-5-uide)

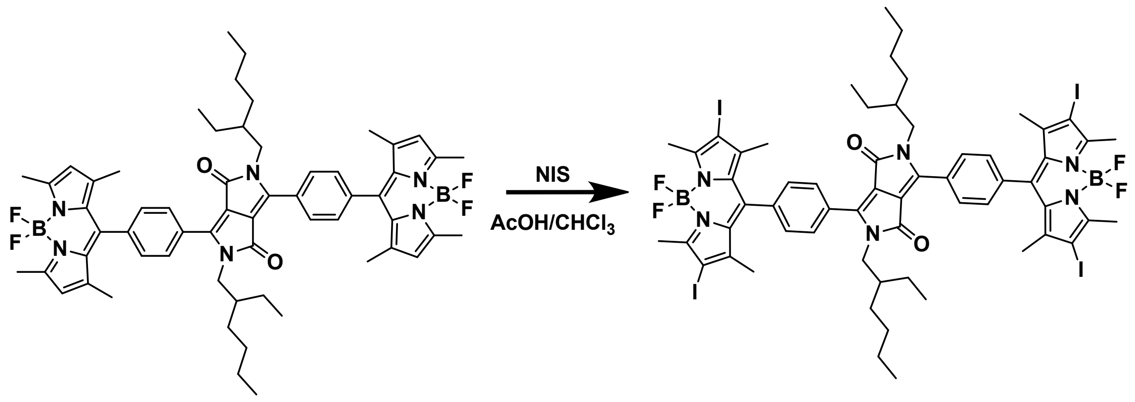

Compound **5** (50.2 mg, 0.5 mmol) was dissolved in a mixture of 18 mL $CHCl_3$/HOAc (3:1), and then NIS (455 mg, 2.5 mmol) was added to the solution, which was reacted for 12 h in

dark. The reaction mixture was washed with water for three times and dried over Na_2SO_4. The solvent was evaporated and the crude product was purified by column chromatography on silica gel with CH_2Cl_2/PE (v/v 1:1) as the eluent. Dark red solid (60.3 mg, 80%). ^{1}H NMR (500 MHz, $CDCl_3$) δ 7.64-7.70 (m, 8H), 3.52 (t, 4H), 2.55 (s, 12H); 1.65-1.50 (m, 36H), 0.10-0.71 (m, 6H); ^{13}C NMR ($CDCl_3$, 500 MHz) δ 162.15, 147.08, 139.40, 135.33, 131.47, 113.96, 109.99, 109.02, 108.02, 107.74, 107.23, 31.76, 29.13, 26.73, 22.62, 13.84. MS (MALDI-TOF) ($[C_{19}H_{17}BF_2N_2I_2]^+$): calcd. m/z = 1508.15; found m/z = 1508.40.

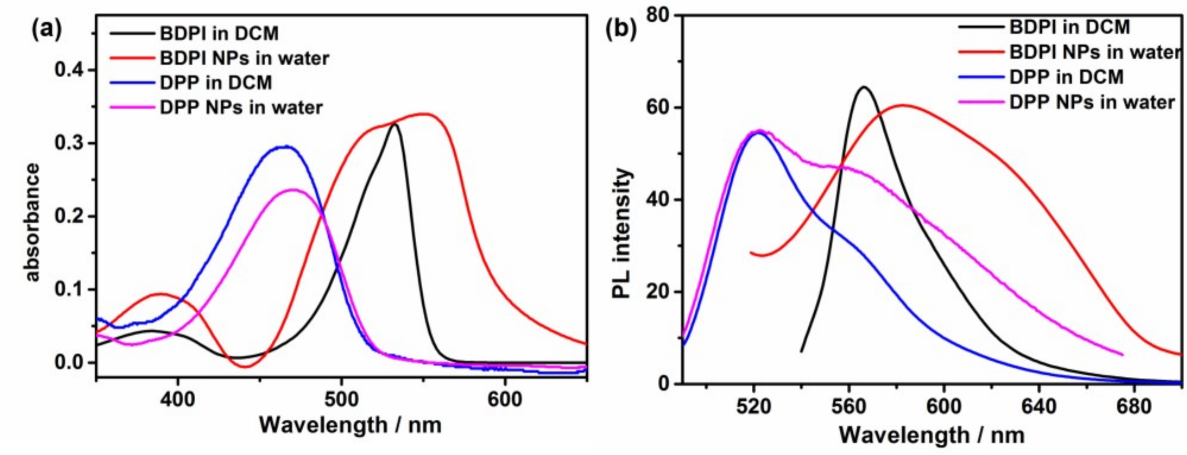

Fig. S2 (a, b) Absorption emission spectra of DPP and BDPI in DCM and their NPs in water.

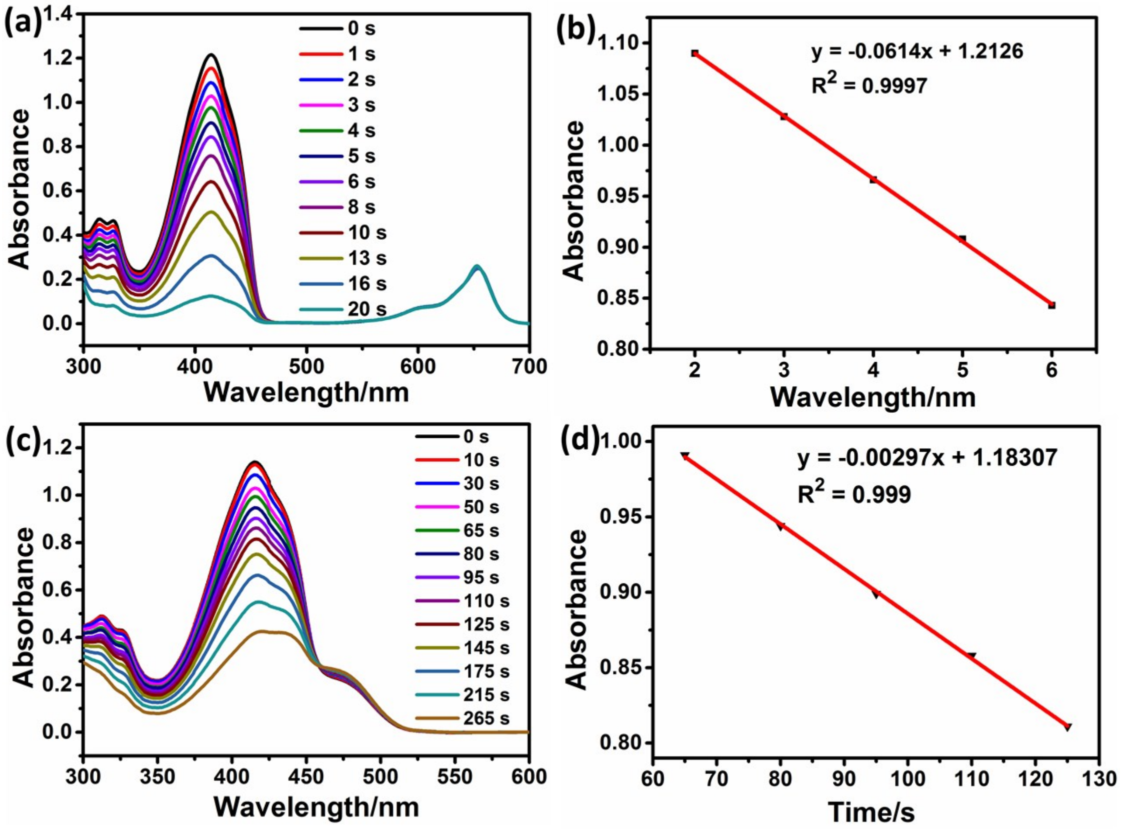

Fig. S3 The degradation of DPBF under Xenon lamp irradiation and linear fitting of the absorbance. (a, b) for methylene blue. (c, d) for DPP

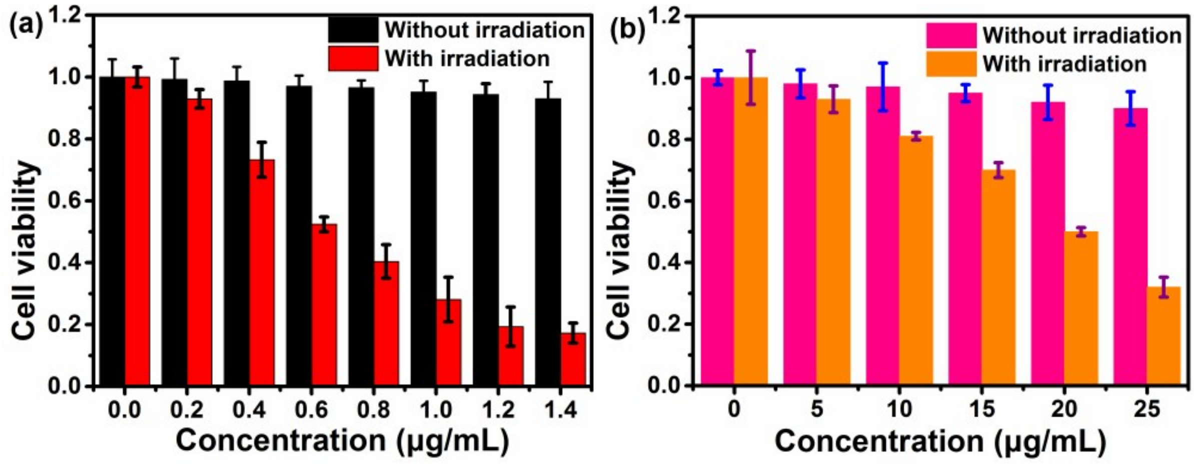

Fig. S4 MTT assay of BDPI, DPP NPs, showing the IC_{50} of 0.6 μg/mL and 22 μg/mL, respectively, (a) for BDPI NPs, (b) for DPP NPs.

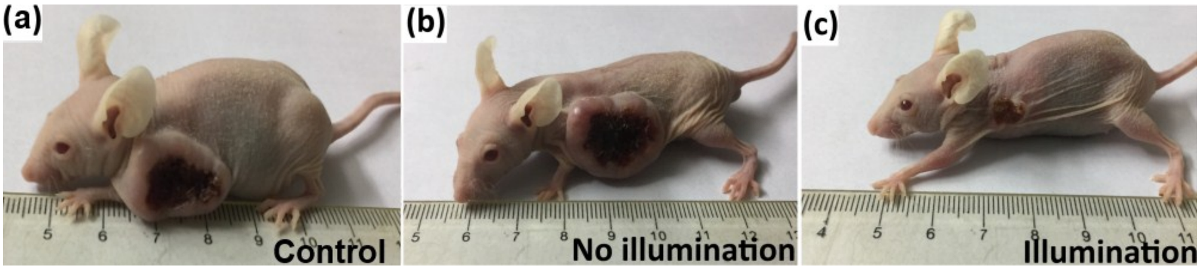

Fig. S5 (a-c) Mice pictures of control, no illumination and illumination group, respectively.
